# *Mo*-CBP_3_, an Antifungal Chitin-Binding Protein from *Moringa oleifera* Seeds, Is a Member of the 2S Albumin Family

**DOI:** 10.1371/journal.pone.0119871

**Published:** 2015-03-19

**Authors:** José E. C. Freire, Ilka M. Vasconcelos, Frederico B. M. B. Moreno, Adelina B. Batista, Marina D. P. Lobo, Mirella L. Pereira, João P. M. S. Lima, Ricardo V. M. Almeida, Antônio J. S. Sousa, Ana C. O. Monteiro-Moreira, José T. A. Oliveira, Thalles B. Grangeiro

**Affiliations:** 1 Departamento de Bioquímica e Biologia Molecular, Centro de Ciências, Universidade Federal do Ceará, Fortaleza, Ceará, Brazil; 2 Núcleo de Biologia Experimental, Universidade de Fortaleza, Fortaleza, Ceará, Brazil; 3 Departamento de Biologia, Centro de Ciências, Universidade Federal do Ceará, Fortaleza, Ceará, Brazil; 4 Instituto de Medicina Tropical (IMT-RN), Universidade Federal do Rio Grande do Norte, Natal, Rio Grande do Norte, Brazil; Henan Agricultural Univerisity, CHINA

## Abstract

*Mo*-CBP_3_ is a chitin-binding protein from *M*. *oleifera* seeds that inhibits the germination and mycelial growth of phytopathogenic fungi. This protein is highly thermostable and resistant to pH changes, and therefore may be useful in the development of new antifungal drugs. However, the relationship of MoCBP3 with the known families of carbohydrate-binding domains has not been established. In the present study, full-length cDNAs encoding 4 isoforms of *Mo*-CBP_3_ (*Mo*-CBP_3_-1, *Mo*-CBP_3_-2, *Mo*-CBP_3_-3 and *Mo*-CBP_3_-4) were cloned from developing seeds. The polypeptides encoded by the *Mo*-CBP_3_ cDNAs were predicted to contain 160 (*Mo*-CBP_3_-3) and 163 amino acid residues (*Mo*-CBP_3_-1, *Mo*-CBP_3_-2 and *Mo*-CBP_3_-4) with a signal peptide of 20-residues at the N-terminal region. A comparative analysis of the deduced amino acid sequences revealed that *Mo*-CBP_3_ is a typical member of the 2S albumin family, as shown by the presence of an eight-cysteine motif, which is a characteristic feature of the prolamin superfamily. Furthermore, mass spectrometry analysis demonstrated that *Mo*-CBP_3_ is a mixture of isoforms that correspond to different mRNA products. The identification of *Mo*-CBP_3_ as a genuine member of the 2S albumin family reinforces the hypothesis that these seed storage proteins are involved in plant defense. Moreover, the chitin-binding ability of *Mo*-CBP_3_ reveals a novel functionality for a typical 2S albumin.

## Introduction

*Moringa*, the only genus of the flowering plant family Moringaceae, comprises 13 species ranging from small herbs to large trees distributed in tropical and subtropical regions. The drumstick tree (*M*. *oleifera* Lam.), also known as the horseradish tree, is a drought-resistant species that is native to northwestern India and is now cultivated in many areas. This species has been described as a multipurpose tree because of its many uses and potential applications. The seeds of *M*. *oleifera*, for example, possess coagulant and antimicrobial agents that have been explored for their ability to treat water and wastewater [1]. The active component or components responsible for these coagulant and antimicrobial effects of *M*. *oleifera* seed extracts have been under investigation since the 1990s. Most previous studies support the hypothesis that cationic peptides and small basic proteins are the active molecules [2–6], although the involvement of an organic 3-kDa polyelectrolyte of unknown structure [7] or other as-yet unrevealed active agents is a possibility.

Recently, a novel chitin-binding protein (CBP) was purified from the seeds of *M*. *oleifera* and named *Mo*-CBP_3_ [8]. *Mo*-CBP_3_ is a 14-kDa thermostable antifungal protein that inhibits the spore germination and mycelial growth of the ascomycete *Fusarium solani* and other fungi [8], [9]. This protein may be useful in the development of new antifungal drugs or transgenic crops with enhanced resistance to phytopathogenic fungi. Although the mechanism of action of *Mo*-CBP_3_ and many other chitin-binding proteins is not fully understood, the antifungal activity of these CBPs is likely the result of protein binding to nascent fungal cell wall chitin, as demonstrated for AFP1, a chitin-binding protein from *Streptomyces tendae* [10].

Carbohydrate-binding modules (CBMs) and lectins are the main classes of carbohydrate-recognizing proteins described to date. CBMs are non-catalytic polysaccharide-recognizing domains that typically occur within multi-modular carbohydrate-active enzymes [11], although in some rare cases, they are found as independent units. For example, Ole e 10 is a 10-kDa pollen protein found in the olive tree (*Olea europaea*) that preferentially binds 1,3-β-glucan and comprises an independent CBM not linked to a catalytically active module [12]. Sixty-nine families of structurally-related CBMs are currently defined in the carbohydrate-active enzymes database (CAZy) [13], and 12 out of the 69 CBM families are known to include members with chitin-binding activity. Lectins are a heterogeneous group of proteins that possess one (merolectins) or two or more (hololectins) non-catalytic domains that bind specifically to a monosaccharide or oligosaccharide [14]. The chimerolectins constitute a third type of lectin in which one or more carbohydrate-binding domains (CBDs) are fused to unrelated domains (not necessarily a carbohydrate-active catalytic domain). Lectins occur as families of structurally and evolutionary related proteins, and some of these families characteristically possess a sugar-binding specificity for oligomers of *N*-acetylglucosamine and chitin, such as those containing the Nictaba or hevein domain [15].

Based on the ability of *Mo*-CBP_3_ to bind chitin, the primary motivation of the present study was to investigate the possible relationship of this protein with any classified CBM or lectin family or to determine whether this novel CBP defines a new CBM or lectin family. Cloning of full-length cDNAs and analysis of the deduced amino acid sequences showed that, contrary to any previous expectations, *Mo*-CBP_3_ is a typical member of the 2S albumin family, which is one of the main classes of seed storage proteins.

## Materials and Methods

### Plant material

Developing seeds of *M*. *oleifera* at 65 days after anthesis were harvested from trees naturally growing at the Campus do Pici, Fortaleza, Ceará, Brazil. Voucher specimens (EAC 54112) were deposited at the Herbário Prisco Bezerra, Universidade Federal do Ceará. Because *M*. *oleifera* is an introduced species that is not native to Brazil, specific permissions from local authorities to obtain the samples used in the present work were not required. The field studies did not involve endangered or protected species. Once harvested, immature seeds were frozen in liquid nitrogen and stored at −80°C until use.

### Plasmids, bacterial strains and reagents

The plasmid pGEM-T Easy was purchased from Promega (Madison, WI, USA), whereas the *Escherichia coli* cloning strain TOP10F′ was from Invitrogen (Carlsbad, CA, USA). All other reagents were of analytical grade.

### Nucleic acid purification

Total RNA was isolated using the Concert Plant RNA Reagent (Invitrogen) according to the manufacturer’s instructions. The integrity of the RNA samples was determined by 1% agarose gel electrophoresis, and the yield was estimated by measuring the absorbance at 260 nm [16]. Prior to cDNA synthesis, total RNA was treated with RQ1 RNase-free DNase I (Promega) at 37°C for 30 min (1 U of DNase I per μg of RNA) and cleaned up using the RNeasy Mini kit (Qiagen, Hilden, Germany). Treated RNA was recovered in 30 μL of nuclease-free water and used for cDNA synthesis.

### 3′ RACE

Total RNA was reverse-transcribed to DNA using the ImProm-II Reverse Transcription System (Promega) and oligo(dT)_18_ primer (Promega) according to the protocol supplied by the manufacturer. The first-strand cDNA products were then submitted to amplification (3′ RACE) using a gene-specific primer (5′-CCGTGYCCGGCNATHCAGCGTTGCT-3′) and oligo(dT)_18_. The gene-specific primer was designed taking into account the N-terminal amino acid sequence determined from the mature *Mo*-CBP_3_ [8]. Amplifications were performed in a final volume of 20 μL, which contained first-strand cDNA (640 ng), 1× GoTaq reaction buffer (Promega), 1.5 mM MgCl_2_, 200 μM each dNTP, 0.5 μM each primer, and 1.25 U GoTaq DNA Polymerase (Promega). The reactions were performed in a PTC-200 thermocycler (MJ Research, USA) using the following cycling parameters: an initial denaturation step (2 min at 95°C) followed by 27 cycles of 1 min at 95°C, 40 s at 52°C, and 30 s at 72°C. After the last cycle, the reactions were further incubated for 5 min at 72°C and stored at −20°C until use.

### 5′ RACE

5′ RACE was performed using the FirstChoice RLM-RACE Kit (Ambion Life Technologies, Carlsbad, CA, USA) following the manufacturer’s protocol with minor modifications. Briefly, total RNA (10–15 μg) was treated initially with calf intestinal alkaline phosphatase (CIP) and subsequently with tobacco acid pyrophosphatase (TAP); both reactions were performed at 37°C for 1 h. The 5′ RACE adapter (5′-GCUGAUGGCGAUGAAUGAACACUGCGUUUGCUGGCUUUGAUGAAA-3′) was then ligated to the CIP/TAP-treated RNA using T4 RNA ligase (37°C for 1 h) and used in reverse transcription. cDNA synthesis was performed using M-MLV reverse transcriptase and random decamers at 50°C for 1 h. Next, the 5′ end of the transcript encoding *Mo*-CBP_3_ was amplified by PCR using the 5′ RACE outer primer (5′-GCTGATGGCGATGAATGAACACTG-3′) and a gene-specific reverse primer. Three distinct reverse primers were used, and these primers were designed based on the sequence information obtained from the 3′ RACE products. The sequences of these primers were as follows: 5′-CACGGGGTACATTTGAGCAACTAGC-3′ (gene-specific reverse primer 1, GSRP1), 5′-AGCTTCGAGCTCTACGAACACACAC-3′ (GSRP 2), and 5′-GTTACACCGCTAGTGGCTCTCGTCT-3′ (GSRP 3). The amplifications were performed in a final volume of 50 μL, which contained first-strand cDNA (640 ng), 1× Green GoTaq reaction buffer (Promega), 1.5 mM MgCl_2_, 200 μM each dNTP, 0.5 μM each primer, and 1.25 U GoTaq DNA Polymerase (Promega). The reactions were carried out in a Mastercycler gradient thermocycler (Eppendorf, Hamburg, Germany) using the following cycling parameters: an initial denaturation step (5 min at 95°C) followed by 33 cycles of 1 min at 95°C, 1.5 min at 60°C, and 1.5 min at 72°C. After the last cycle, the reactions were further incubated for 15 min at 72°C and stored at −20°C.

### Cloning of PCR products

The specificity of the PCR amplifications (5′ and 3′ RACE) and the sizes of the amplicons were determined by 1% agarose gel electrophoresis [16]. An aliquot of the remaining amplified products was ligated into the pGEM-T Easy vector using T4 DNA ligase (Promega) at 4°C for 16 h. Products from the ligation reactions were introduced into *E*. *coli* TOP10F′ cells by electroporation, and the transformants were selected on LB agar containing 100 μg.mL^-1^ carbenicillin and 30 μg.mL^-1^ streptomycin. Plasmid DNA was isolated from antibiotic-resistant colonies using the alkaline lysis method [16], and the presence of the inserts was confirmed by restriction digestion with *Eco*RI (Fermentas Life Sciences, Ontario, Canada).

### DNA sequencing and assembly

Plasmid samples for DNA sequencing were purified using the AxyPrep plasmid miniprep kit (Axygen Scientific, Union City, CA, USA). The inserts were sequenced using the DYEnamic ET Dye terminator cycle sequencing kit (GE Healthcare, Buckinghamshire, UK) following the protocol supplied by the manufacturer. Both strands were sequenced using the universal primers M13 (-40) forward (5′-GTTTTCCCAGTCACGACGTTGTA-3′) and M13 (-46) reverse (5′-GAGCGGATAACAATTTCACACAGG-3′). The sequencing products were resuspended in 10 μL of 70% formamide/1 mM EDTA, and prior to capillary electrophoresis, 10 μL of agarose was added (0.06% final concentration) as suggested previously [17], [18]. The sequencing reactions were analyzed in a MegaBACE 1000 automatic sequencer (GE Healthcare) using the following parameters: injection at 3 kV for 50 s and electrophoresis at 6 kV for 200 min. Automated base-calling was performed using Cimarron 3.12 software, and complete sequences were assembled using the Phred/Phrap/Consed package [19–21]. Before further analysis, the ends of the assembled contigs were trimmed to remove low-quality (phred < 20) sequences.

### Sequence analysis

Multiple alignments of DNA and amino acid sequences were usually performed using the program Clustal W [22] implemented with the BioEdit 7.2.5 software package [23], which was routinely used for sequence manipulation, editing and comparisons. On the other hand, the 3ʹ UTR sequences were aligned using the program Clustal Omega [24] at the web server www.ebi.ac.uk/Tools/msa/clustalo/. The default alignment parameters of Clustal Omega were employed, although the number of combined iterations and the maximum number of HMM iterations were both set to five. Codon-based alignments were performed using the program PAL2NAL [25] at the program’s web server (www.bork.embl.de/pal2nal/). The identity between two aligned sequences was calculated as the number of positions containing identical nucleotides or amino acid residues divided by the number of aligned positions, excluding the sites with gaps, and expressed as a percentage. RNA secondary structures were predicted using Vienna RNA Secondary Structure Prediction version 2.1.6 (http://rna.tbi.univie.ac.at/cgi-bin/RNAfold.cgi.) [26]. The presence of signal peptides and their putative cleavage sites were predicted using the algorithm SignalP 4.1 (www.cbs.dtu.dk/services/SignalP/) [27]. Searches for homologous proteins in public sequence databases were performed using BLASTp [28]. The presence and delimitation of protein domains was accomplished using the Conserved Domain Database (CDD) [29].

### Phylogenetic analysis

Phylogenetic analysis was performed using Molecular Evolutionary Genetics Analysis (MEGA) software version 6.0 [30]. The amino acid sequences of the proteins were aligned using Clustal Omega, and the pairwise evolutionary distances were then computed using the Poisson correction method [31]. The trees were generated using the neighbor-joining method [32], and the stability of the clades was assessed using the bootstrap method [33].

### Purification of *Mo*-CBP_3_

*Mo*-CBP_3_ was purified from crude extracts of mature *M*. *oleifera* seeds using affinity chromatography on a chitin matrix followed by cation exchange chromatography on a Resource S matrix (GE Healthcare) as described previously [8]. The purity of the protein samples was determined by tricine-SDS-polyacrylamide gel electrophoresis (tricine-SDS-PAGE) according to a previously described method [34]. Protein bands were stained with 0.1% (w/v) Coomassie Brilliant Blue R250 in 40% methanol/1% acetic acid. Destaining was carried out with 50% (v/v) methanol.

### N-terminal amino acid sequencing

N-terminal sequencing was performed on a Shimadzu PPSQ-10 Automated Protein Sequencer (Kyoto, Japan). Protein samples were blotted onto a polyvinylidene fluoride (PVDF) membrane after tricine-SDS-PAGE and submitted to Edman degradation [35]. The phenylthiohydantoin (PTH) amino acids were detected at 269 nm after separation on a reverse-phase C18 column (4.6 mm x 2.5 mm) under isocratic conditions according to the manufacturer’s instructions.

### Capillary liquid chromatography/nanoelectrospray ionization tandem mass spectrometry (LC-ESI-MS/MS)

In-gel tryptic digestions of proteins bands resolved by tricine-SDS-PAGE were performed according to a protocol described previously [36]. Protein samples were also submitted to in-solution digestions. To this end, the samples were reduced with 5 mM DTT at 60°C for 30 min, treated with 15 mM iodoacetamide at room temperature for 30 min in the dark, and digested with sequencing-grade trypsin (Promega) at 37°C for 16 h. The tryptic peptides from in-gel and in-solution digestions were analyzed by LC-ESI-MS/MS using a Synapt G1 HDMS Q-ToF mass spectrometer (Waters Co., Milford, MA, USA) coupled to a Waters ultra-high-performance liquid chromatography (UPLC) unit. The digested samples were injected using the nanoACQUITY UPLC sample manager, and the chromatography was performed using a UPLC C18 nanoACQUITY column (75 μm x 10 cm, 1.7 μm particle size) at a flow rate of 0.35 μL/min. The mass spectra were acquired using the data-dependent acquisition (DDA) mode, in which the top three peaks were subjected to MS/MS. Mobile phases A and B consisted of 0.1% formic acid in water and 0.1% formic acid in acetonitrile, respectively. The peptides were eluted using the following step gradient: 3–40% B for 30 min and 40–85% B for 5 min. The data were processed using Protein Lynx Global Server software (Waters Co.) and subjected to a database search using the Mascot search engine [37]. The searches were performed with the assumptions that there was a maximum of one missed trypsin cleavage and the experimental masses of the peptides were monoisotopic. Furthermore, carbamidomethylation of cysteine was included as a fixed modification, whereas oxidation of methionine and cyclization of N-terminal glutamine to pyroglatamic acid (pyro-Glu) were included as possible variable modifications. MS/MS ions searches were performed against the NCBI non-redundant database (last accessed on January 21st, 2015) using a significance threshold of *p* < 0.05. The peptide mass tolerance and fragment mass tolerance were both initially set to ± 0.1 Da for MS/MS ion searching. However, candidate peptide IDs were only accepted if the *m/z* values were within 0.1 Da (typically less than 0.05 Da) of the theoretical mass of the candidate ID, as determined when manually reviewing the MASCOT search results. The mass spectrometry proteomics data have been deposited to the ProteomeXchange Consortium [38] via the PRIDE partner repository with the dataset identifier PXD001762 and null.

## Results

### RACE and cDNA assembly

**Fig 1:**
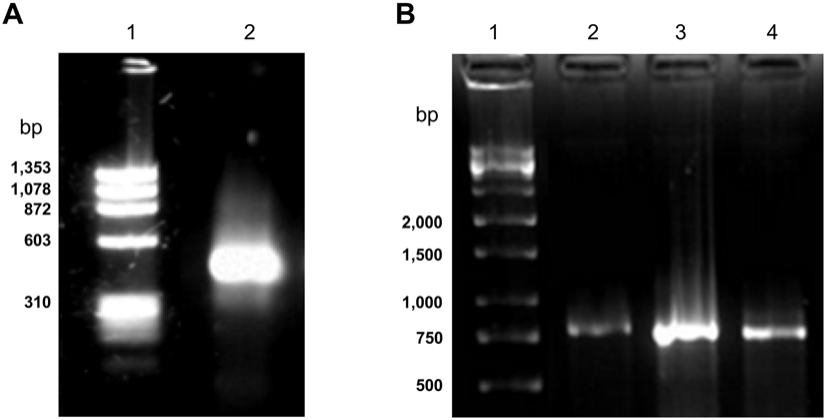
Agarose gel electrophoresis of *Mo*-CBP_3_ cDNA fragments amplified by PCR. A. 3′ RACE products (lane 1). B. 5′ RACE products amplified using 3 distinct gene-specific primers (lanes 1, 2 and 3). Lane M (A and B): molecular markers.

Using a combination of 5′ RACE and 3′ RACE, full-length cDNAs encoding chitin-binding protein 3 from *M*. *oleifera* (*Mo*-CBP_3_) were obtained from developing seeds. The 3′ end of the *Mo*-CBP_3_ mRNA was amplified using oligo(dT) as an antisense primer and a gene-specific degenerate oligonucleotide as a sense primer; this primer was designed by referencing the N-terminal sequence of the purified protein [8]. Agarose gel electrophoresis of the 3′ RACE products revealed a single DNA band of approximately 420 bp (Fig. 1A). The amplified cDNA fragment was cloned, and the inserts of 20 clones were completely sequenced. When these sequences were aligned and compared, it was possible to cluster them into 3 groups according to their overall similarity. Therefore, gene-specific oligonucleotides targeting each one of these 3 distinct 3′ untranslated regions (UTRs) were designed and used as reverse primers in the 5′ RACE experiment. An oligonucleotide targeting the RNA adapter, which was ligated to the 5′ ends of the total mRNAs, was used as a forward primer. Agarose gel electrophoresis of the 5′ RACE products showed that 3 specific amplicons were produced, with estimated sizes of approximately 790, 800 and 780 bp (Fig. 1B). These PCR products were cloned, and the complete sequences of the inserts from 42 clones were determined.

**Fig 2:**
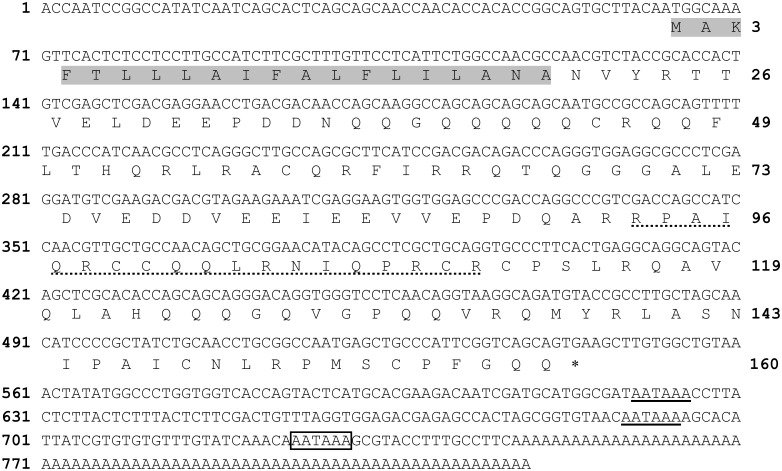
The cDNA sequence and deduced amino acid sequence of prepro*Mo*-CBP_3_–3. The deduced amino acid sequence of the prepro*Mo*-CBP_3_–3 is shown below the cDNA sequence. Numbers for the first nucleotide and the last amino acid residue in each row are shown on the left and right, respectively. The N-terminal signal peptide, as predicted by the SignalP 4.1 program [27], is shaded in gray. The N-terminal sequence of the large chain of *Mo*-CBP_3_, as determined by Edman degradation, is underlined with a dashed line. The stop codon is indicated by an asterisk. The poly(A) signal nearest to the poly(A) tail is boxed, and two other upstream poly(A) signals are underlined.

The overlapping DNA sequences obtained from all cloned PCR products from both the 3′ RACE and 5′ RACE reactions were assembled. As a result, 4 unique cDNA contigs presumptively encoding *Mo*-CBP_3_ were generated. Herein, these cDNA sequences (GenBank accession numbers KF616830-KF616833) are referred to as *Mo-CBP*_*3*_–*1* (695 bp), *Mo-CBP*_*3*_–*2* (797 bp), *Mo-CBP*_*3*_–*3* (819 bp), and *Mo-CBP*_*3*_–*4* (827 bp) according to their lengths. Three of these assembled fragments, *Mo-CBP*_*3*_–*2*, *Mo-CBP*_*3*_–*3*, and *Mo-CBP*_*3*_–*4*, corresponded to full-length cDNAs, as each one contained complete 5′ and 3′ UTRs and a single coding sequence (CDS). The fourth contig (*Mo-CBP*_*3*_–*1*) contained a near-full-length cDNA, with a complete 5′ UTR and CDS but with a partial 3′ UTR (Table 1). The full-length cDNA sequence of *Mo-CBP*_*3*_–*3* and its deduced amino acid (aa) sequence are shown in Fig. 2 as a representative sequence of the 4 assembled cDNAs.

**Table 1:** General features of the cDNA sequences encoding *Mo*-CBP_3_.

			cDNA regions			
cDNA	GenBank accession number	Length (nt)	5′ UTR (nt)	CDS (nt)/preproprotein size (aa)	3′ UTR (nt)	Poly(A) site
*Mo*-CBP_3_–1	KF616830	695	1–56	57–548 (492)/163	549–695 (147)^1^	n.d.^2^
*Mo-*CBP_3_–2	KF616832	797	1–56	57–548 (492)/163	549–751 (203)	752
*Mo-*CBP_3_–3	KF616833	819	1–62	63–545 (483)/160	546–747 (202)	748
*Mo-*CBP_3_–4	KF616831	827	1–56	57–543 (487)/163	544–747 (204)	748

^1^ Partial 3′ UTR sequence

^2^ Not determined

Pairwise comparisons of the 4 cDNA sequences revealed an overall mean sequence identity of approximately 82.8% (excluding all sites with insertions/deletions). The cDNA sequences of *Mo-CBP*_*3*_–*1* and *Mo-CBP*_*3*_–*4* were very closely related to each other (99.3% pairwise sequence identity), whereas the sequence of *Mo-CBP*_*3*_–*3* was the most divergent from the other sequences (average pairwise sequence identity of approximately 78.1%).

### The CDS and the 5′ and 3′ UTRs

Within each cDNA sequence, one open reading frame (ORF) was found in frames 1 (*Mo-CBP*_*3*_–*2*) and 3 (*Mo-CBP*_*3*_–*1*, *Mo-CBP*_*3*_–*3* and *Mo-CBP*_*3*_–*4*). The ORF size varied from 483 nucleotides (nt) (in *Mo-CBP*_*3*_–*3*) to 492 nt (in *Mo-CBP*_*3*_–*1*, *Mo-CBP*_*3*_–*2* and *Mo-CBP*_*3*_–*4*), as summarized in Table 1. The average sequence identity among the CDSs was ~88%, ranging from 83.9% (*Mo-CBP*_*3*_–*1* and *Mo-CBP*_*3*_–*3*) to 99.3% (*Mo-CBP*_*3*_–*1* and *Mo-CBP*_*3*_–*4*). In these ORFs, the context sequence around the ATG start codon (AUG in the mRNA) was in agreement with the consensus sequence AAAA/CAAUGGC of the translational initiation site (TIS), which was derived from the analysis of 3643 plant genes [39]. Therefore, the following sequences were found for the segment spanning the nucleotide positions from −5 (immediately upstream) to +5 (immediately downstream of the ATG start codon): TTACTatgGC (*Mo-CBP*_*3*_–*1* and *Mo-CBP*_*3*_–*4*), TTACCatgGC (*Mo-CBP*_*3*_–*2*), and TTACAatgGC (*Mo-CBP*_*3*_–*3*) (nucleotides that match those found in the consensus sequence of the TIS are underlined). The A and G nucleotides at positions −3 and +4 around the ATG start codon, as observed in the 4 *Mo-CBP*_*3*_ cDNA sequences, have been suggested to be particularly important for greater translational efficiency [40], [41].

**Fig 3:**
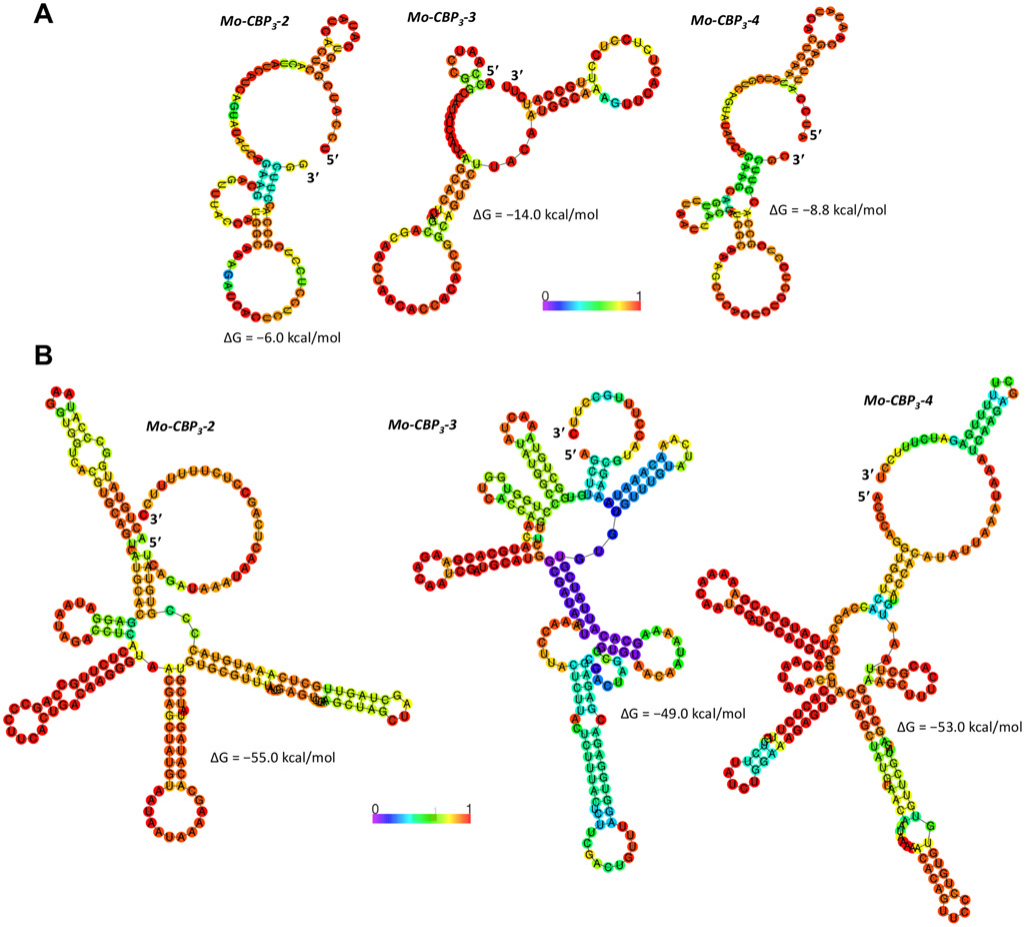
Predicted secondary structures of the 5′ UTR and 3′ UTR sequences of the *Mo-CBP*_3_ mRNAs. A. The predicted MFE secondary structures of the entire 5′ UTR and the first 32 nt of the CDS of the *Mo-CBP*_*3*_–*2*, *Mo-CBP*_*3*_–*3* and *Mo-CBP*_*3*_–*4* mRNAs are shown. B. The predicted MFE secondary structures of the entire 3′ UTRs of the *Mo-CBP*_*3*_–*2*, *Mo-CBP*_*3*_–*3* and *Mo-CBP*_*3*_–*4* mRNAs are shown. Heat color gradation from blue to red represents the base-pairing probability from 0 to 1.

The 5′ UTRs in the *Mo-CBP*_*3*_ cDNAs were 51 (*Mo-CBP*_*3*_–*2*), 56 (*Mo-CBP*_*3*_–*1* and *Mo-CBP*_*3*_–*4*) and 62 (*Mo-CBP*_*3*_–*3*) nt long. The sequence identity among these UTRs was 80% on average, varying from 100% (5′ UTRs of *Mo-CBP*_*3*_–*1* and *Mo-CBP*_*3*_–*4*) to 66.6% (5′ UTRs of *Mo-CBP*_*3*_–*1 vs Mo-CBP*_*3*_–*3* and of *Mo-CBP*_*3*_–*3 vs Mo-CBP*_*3*_–*4*). The lengths of the 5′ UTRs of the *Mo-CBP*_*3*_ cDNAs thus fall within the size range that was reported for this region in a survey of 1,615 Viridiplantae genes, which were shown to have 5′ UTRs that are 116 nt long on average [42]. The entire 5′ UTR and the first 32 nt of the CDS of the *Mo-CBP*_*3*_ sequences were predicted to fold into a secondary structure characterized by 2 or 3 hairpins radiating from a central loop (Fig. 3A). The ΔG of the minimum free energy (MFE) secondary structures ranged from −14.0 kcal/mol to −6.0 kcal/mol, suggesting that even under the assumption that these structures could occur *in vivo*, they would not be sufficiently stable enough to inhibit translation [43].

The 3′ UTR sequences of the *Mo-CBP*_*3*_ cDNAs were longer and more divergent than the sequences of the 5′ UTRs. Their lengths (excluding the segments of the poly(A) tail) were 203 (*Mo-CBP*_*3*_–*4*), 204 (*Mo-CBP*_*3*_–*2*) and 202 nt (*Mo-CBP*_*3*_–*3*). The partial sequence obtained for the 3′ UTR of *Mo-CBP*_*3*_–*1* was 147 nt long. The mean sequence identity among the *Mo-CBP*_*3*_ 3′ UTRs was approximately 67.7%, ranging from 60.2% (3′ UTR of *Mo-CBP*_*3*_–*2 vs Mo-CBP*_*3*_–*4*) to 99.2% (3′ UTR of *Mo-CBP*_*3*_–*1 vs Mo-CBP*_*3*_–*4*). The lengths of the *Mo-CBP*_*3*_ 3′ UTRs are comparable to the average length (~ 240 nt) of the 3′ UTRs of 1,826 Viridiplantae genes [42]. In the 3′ UTRs of *Mo-CBP*_*3*_–*3* and *Mo-CBP*_*3*_–*4*, a typical eukaryotic polyadenylation signal sequence (AATAAA) was found 16 and 25 nt upstream of the first A of the poly(A) tail, respectively (Fig. 2). In the 3′ UTR of *Mo-CBP*_*3*_–*2*, a close variant (GATAAA) of the canonical polyadenylation signal sequence was observed 20 nt upstream of the poly(A) tail. Furthermore, in the 3′ UTRs of *Mo-CBP*_*3*_–*2* and *Mo-CBP*_*3*_–*3*, the first A of the poly(A) tail is preceded by C, whereas in the 3′ UTR of *Mo-CBP*_*3*_–*4*, the poly(A) tail is preceded by T. This agrees with the finding that in plant genes, cleavage of the pre-mRNA during 3′-end processing usually occurs 3′ to an adenosine residue at the dinucleotide YA (Y = C or T) [44]. The full-length 3′ UTRs of the *Mo-CBP*_*3*_ transcripts were predicted to be able to fold into stable secondary structures (Fig. 3B). The calculated ΔG of the MFE secondary structures ranged from −49.0 kcal/mol to −55.0 kcal/mol. The 5′ and 3′ UTRs of many mRNAs characteristically contain *cis*-regulatory elements that play crucial roles in post-transcriptional regulation. Many of these cis-regulatory elements are structured, and they influence distinct aspects of the mRNA life cycle such as stability, transport, localization, and translation activation and repression [45–47]. The secondary structures predicted to occur in the UTRs of *Mo-CBP*_*3*_ mRNAs could play similar roles.

Therefore, these sequence analyses demonstrated that the *Mo-CBP*_*3*_ cDNAs have the general structural features usually found in plant genes.

### The proteins encoded by the *Mo-CBP*_*3*_ cDNAs

The polypeptides encoded by the *MoCBP*_*3*_ cDNAs were predicted to contain 160 (*Mo*-CBP_3_–3) and 163 aa residues (*Mo*-CBP_3_–1, *Mo*-CBP_3_–2 and *Mo*-CBP_3_–4). The percentage sequence identity among the 4 putative primary translation products was ~81% on average, ranging from 76.4% (between the products of *Mo-CBP*_*3*_–*1* and *Mo-CBP*_*3*_–*3*) to 98% (between the products of *Mo-CBP*_*3*_–*1* and *Mo-CBP*_*3*_–*4*), indicating that the encoded polypeptides are closely related to each other. When these sequences were analyzed with SignalP software, the segment comprising the first 20 aa residues of each protein was predicted to be a signal peptide (SP). The SPs of the 4 encoded proteins have unique aa sequences, and the average sequence identity between them is approximately 77.5%. Each *Mo*-CBP_3_ SP has the classical tripartite structure comprising a positively charged N-terminal region (3 aa residues long, containing a Lys residue) followed by a central hydrophobic core (13 aa residues long, with a high proportion of Leu residues) and a neutral but polar C-terminal region (4 aa residues long) containing the putative signal peptidase (SPase) cleavage site. Positions −1 and −3 relative to the SPase cleavage site are occupied by Ala and Ala (in 3 sequences) or Ala and Thr (in one sequence), respectively; this finding agrees with the specificity of SPase cleavage site [48]. Indeed, seed storage proteins are typically synthesized with an N-terminal SP that is cleaved as the proteins are translocated into the lumen of the endoplasmic reticulum where they are subjected to post-translational processing and then transported to protein storage vacuoles [49].

**Fig 4:**
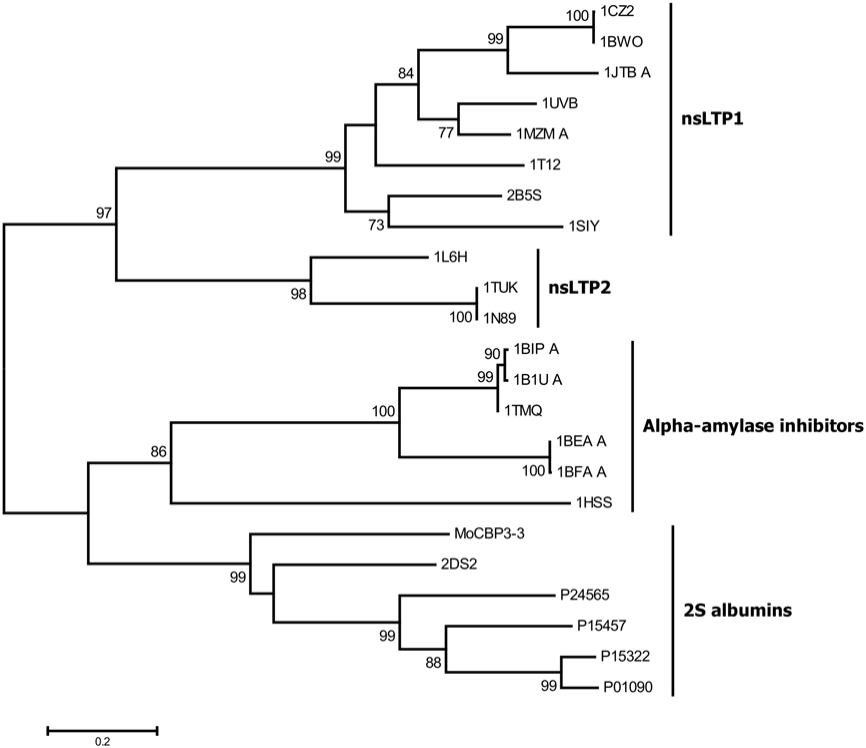
Unrooted neighbor-joining (NJ) tree depicting the phylogenetic relationship of *Mo*-CBP_3_–3 with representative members of the prolamin superfamily. The amino acid sequences of the small and large chains of *Mo*-CBP_3_–3 (this work) were concatenated and aligned to the corresponding sequences of representative non-specific type 1 LTP (nsLTP1), type 2 LTP (nsLTP2), alpha-amylase inhibitors and 2S albumins. The evolutionary distances were computed using the Poisson correction method, and the units are the number of amino acid substitutions per site. The percentages of replicate trees in which the associated sequences clustered together in the bootstrap test (1000 replicates) are shown next to the branches. The PDB codes or GenBank accession numbers of the sequences used are shown.

To further clarify the identity of the proteins encoded by the *Mo-CBP*_*3*_ cDNAs, the deduced aa sequences of their putative precursors (pro*Mo*-CBP_3_, excluding their N-terminal SPs) were submitted to BLAST searches against protein databases. The searches against the Conserved Domain Database revealed that each pro*Mo*-CBP_3_ sequence possesses a single AAI_LTSS domain (CDD superfamily accession number: cl07890), which is characteristic of the prolamin superfamily. This superfamily is unique to higher plants and includes i) cereal-type α-amylase inhibitors (AAI), trypsin inhibitors, and bifunctional trypsin/ α-amylase inhibitors; ii) lipid transfer proteins (LTPs), such as the non-specific type 1 LTP (nsLTP1) and type 2 LTP (nsLTP2); iii) seed storage (SS) proteins, such as 2S albumins, γ-gliadin, and prolamin; and iv) other related proteins [50], [51]. More specifically, the pro*Mo*-CBP_3_ sequences were clustered in the subfamily AAI_SS (conserved domain model accession number: cd00261), which includes the α-amylase inhibitors and seed storage proteins such as 2S albumins. Searches against the non-redundant protein sequence database of the NCBI using BLASTp revealed that the pro*Mo*-CBP_3_ sequences were more closely related (45–47% sequence identity; E-value = 4×10^-25^–1×10^-19^; 95–97% query coverage) to the aa sequence of a precursor of the sweet protein Mabinlin II, a 2S albumin from the seeds of *Capparis masaikai* [52]. All the other proteins that showed significant alignment with the pro*Mo*-CBP_3_ sequences were 2S seed storage proteins from different species. To further support these findings, a phylogenetic tree was generated using primary structures from representative nsLTP1, nsLTP2, AAIs, and 2S albumins. As shown in Fig. 4, four supported clades were recovered, corresponding to the nsLTP1, nsLTP2, AAIs and 2S albumin subfamilies. The pro*Mo*-CBP_3_–3 aa sequence was confidently placed in the cluster of 2S albumins in this NJ tree.

### The structural features of the *Mo*-CBP_3_ precursors and a possible processing mechanism

**Fig 5:**
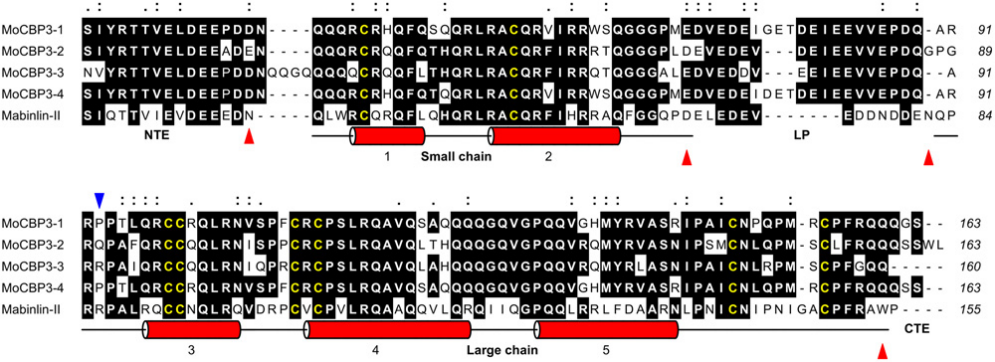
Multiple alignment of the amino acid sequences of the *Mo*-CBP_3_ precursors with proMabinlin-II. The amino acid sequences of the precursors of *Mo*-CBP_3_ were aligned to the sequence of proMabinlin-II (GenBank accession number P30233) using Clustal Omega. Positions containing the same residue in at least 3 sequences are shaded, and the Cys residues are highlighted in yellow. Sites containing residues with side chains that have strongly (:) or weakly (.) similar properties, scoring > 0.5 and ≤ 0.5 in the Gonnet PAM 250 matrix [93], respectively, are also indicated. The α-helices of the small and large chains of Mabinlin-II (PDB code 2DS2) are shown as cylinders. The N-terminal extension (NTE), the linker peptide (LP) and the C-terminal extension (CTE) of the proMabinlin-II are labeled. The processing sites in the proMabinlin-II sequence are indicated by red triangles, whereas the N-terminal residue of the large chain of *Mo*-CBP_3_, as identified by Edman degradation, is indicated by a blue triangle. The numbers of the residues relative to Met^1^ are shown on the right side of each sequence. The alignment was edited using the program ALINE [94].

The aa sequences of the *Mo*-CBP_3_ precursors were 140 (pro*Mo*-CBP_3_–3) and 143 (pro*Mo*-CBP_3_–1, pro*Mo*-CBP_3_–2 and pro*Mo*-CBP_3_–4) residues long. The percentage sequence identity among their primary structures was ~81.6% on average, ranging from 75.7% (between pro*Mo*-CBP_3_–1 and pro*Mo*-CBP_3_–3) to 98.5% (between pro*Mo*-CBP_3_–1 and pro*Mo*-CBP_3_–4). The pro*Mo*-CBP_3_ sequences were easily aligned to the aa sequence of the Mabinlin-II precursor, which has a length of 135 aa residues (Fig. 5). The sequence identity between the precursors of *Mo*-CBP_3_ and the precursor of Mabinlin-II ranged from 48.1% (between pro*Mo*-CBP_3_–2 and proMabinlin-II) to 52.7% (between pro*Mo*-CBP_3_–1/pro*Mo*-CBP_3_–4 and proMabinlin-II). In this multiple alignment, approximately 41.2% of the aligned residues were conserved, and 23.7% of the aligned positions contained residues with chemically similar side chains. Eight conserved Cys residues occur in the primary structures of the *Mo*-CBP_3_ and Mabinlin-II precursors. These Cys residues follow a conserved pattern (…C…C… / …CC…CxC…C…C…), which is called the eight-cysteine motif (8CM). This sequence motif is a characteristic structural feature of all 2S albumins and is also found in other members of the prolamin superfamily [49], [53]. pro*Mo*-CBP_3_ sequences are also rich in Gln (17.5–20.7%), Arg (11.9–13.3%) and Glu (7.1–7.7%), and this bias in the aa composition profile toward polar residues is another characteristic feature observed in many 2S albumins and other seed storage proteins of the prolamin superfamily [53]. Because of the evident structural relationship between pro*Mo*-CBP_3_ and proMabinlin-II, further structural comparisons were performed to provide insights into the processing of the *Mo*-CBP_3_ precursors.

The mature Mabinlin-II is composed of a small chain with 33 aa residues (the A chain) and a large chain with 72 aa residues (the B chain). There are four disulfide bonds two between the A and B chains and two within the B chain and the protein has a total molecular mass of 12.4 kDa [54], [55]. Mabinlin-II is synthesized as a preproprotein with 155 aa residues, which undergoes co- and post-translational processing during seed development. After removal of the 20-aa N-terminal SP, three other segments are further cleaved off from the 135-aa precursor: an N-terminal extension (NTE) peptide of 15 aa residues, a linker peptide (LP) of 14 aa residues located between the small and large chains, and a C-terminal extension (CTE) consisting of one Pro residue [52]. Approximately 40% of the aligned residues in the NTE regions of proMabinlin-II and pro*Mo*-CBP_3_ are conserved, whereas 26.7% of the aligned sites are occupied by residues with similar side chains. In the LP segment, these numbers are 30.8% (aligned sites containing identical residues) and 46.1% (aligned positions occupied by chemically similar residues). Moreover, the NTE and LP regions of proMabinlin-II and the corresponding segments in the pro*Mo*-CBP_3_ sequences are characterized by a significant proportion of hydrophilic residues, especially Glu and Asp (Fig. 5). These hydrophilic propeptides are predicted to be exposed on the molecular surface of the 2S albumin precursors and contain the cleavage sites for vacuolar processing enzymes involved in their maturation [56], [57].

Based on this comparative sequence analysis, it was speculated that *Mo*-CBP_3_ precursors are likely to be processed similarly to proMabinlin-II and other seed storage albumins. However, single chain 2S albumins whose precursors are not cleaved in this manner have also been described, such as SFA-8 from sunflower (*Helianthus annuus*) [58] and Ara h 2 from peanut (*Arachis hypogea*) [59].

**Fig 6:**
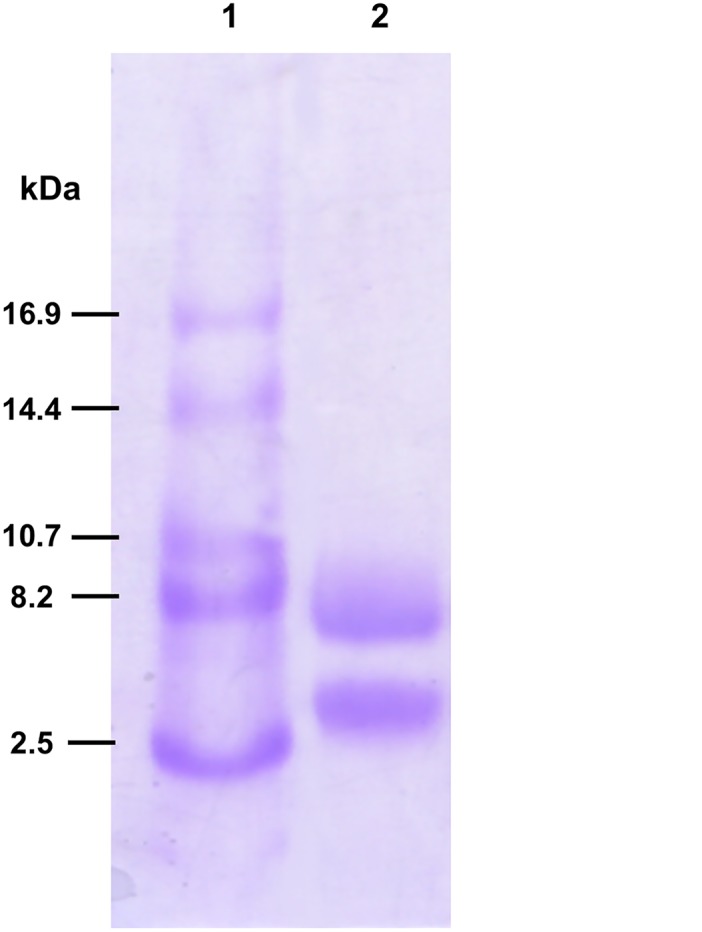
Tricine-SDS-polyacrylamide gel electrophoresis of *Mo*-CBP_3_. *Mo*-CBP_3_ was purified from *M*. *oleifera* seeds using affinity chromatography on a chitin matrix followed by cation exchange chromatography as described previously [8]. Protein bands were resolved by tricine-SDS-PAGE (17.5% polyacrylamide) and stained with Coomassie Brilliant Blue as described in the methods section. Lane 1: molecular weight markers. Lane 2: *Mo*-CBP_3_ treated with β-mercaptoethanol (10 μg per lane).

To verify whether *Mo*-CBP_3_ is a single- or two-chain 2S albumin, the purified protein was treated with β-mercaptoethanol, and the sample was analyzed by tricine-SDS-PAGE. As shown in Fig. 6, two protein bands with apparent molecular masses of approximately 4.1 and 8.1 kDa were detected. Therefore, *Mo*-CBP_3_ is composed of a small 4.1 kDa chain and a large 8.1 kDa chain linked by disulfide bonds. Unreduced *Mo*-CBP_3_ was reported to migrate as a single band with an apparent molecular mass of ~18 kDa when submitted to SDS-PAGE [8]. This anomalous migration was also observed for the unreduced 2S albumins of radish [60]. This atypical mobility can be explained by the observation that disulfide bonds might reduce SDS binding to globular proteins by up to 2-fold [61].

To confirm the identity of the 4.1 and 8.1 kDa polypeptides of *Mo*-CBP_3_, these bands were electroblotted to a PVDF membrane and submitted to N-terminal sequencing. Automated Edman degradation of the small chain failed to yield any identifiable sequence, suggesting that the N-terminal residue was blocked. The cleavage site between the NTE peptide and the small chain that is recognized during the processing of proMabinlin-I is preceded by an Asn residue. In the pro*Mo*-CBP_3_ aa sequences, one can assume that the equivalent processing site would be located at the C-terminal side of Asn^36^, which would result in Gln^37^ becoming the N-terminal residue of the small chain. Cyclization of N-terminal Gln to pyroglutamate (pyro-Glu) leads to a blocked chain, and this event has been described for several 2S albumins [62]. Based on these findings, the length of the small chain of the *Mo*-CBP_3_ isoforms was tentatively determined to be 33 (isoforms 1 and 4: ^37^QQQ···PME^69^; isoform 2: ^37^QQQ···PLD^69^) or 37 (isoform 3: ^37^QQG···ALE^73^) aa residues, assuming that pro*Mo*-CBP_3_ is processed at cleavage sites that are similar to those found for proMabinlin-II. When the 4.1 kDa band resolved by tricine-SDS-PAGE was excised from the gel, digested with trypsin and the products were analyzed by LC-ESI-MS/MS, 4 peptides were identified (Table 2, peptides 4–7). The sequences of these peptides matched exactly with a specific segment in the presumed primary structures of the small chain of *Mo*-CBP_3_–2 (2 peptides) and *Mo*-CBP_3_–3 (2 peptides), thus confirming the identity of the 4.1 kDa band. Therefore, the predicted molecular masses of the small chain were calculated as 4,038.01 (*Mo*-CBP_3_–1), 4,052.09 (*Mo*-CBP_3_–2), 4,410.23 (*Mo*-CBP_3_–3) and 4,052.02 Da (*Mo*-CBP_3_–4).

**Table 2:** Amino acid sequences of *Mo*-CBP_3_ peptides identified by LC-ESI-MS/MS from in-gel tryptic digestions of protein bands separated by tricine-SDS-PAGE.

	Mass (Da)							
Peptide	Experimental	Calculated	Score^1^	Sequence^2^	Modification	Isoform	Chain and coverage (%)^3^	GenBank accession number
1	2099.1096	2099.0984	95	^117^QAVQLAHQQQGQVGPQQVR^135^	-	3	L (27.9)	AHG99684
2	2082.0748	2082.0719	95	^117^ QAVQLAHQQQGQVGPQQVR^135^	Gln→pyro-Glu	3	L (27.9)	AHG99684
3	2129.1385	2129.1090	78	^115^QAVQLTHQQQGQVGPQQVR^133^	-	2	L (27.9)	AHG99683
4	1055.5182	1056.5465	58	^47^QQFLTHQR^54^	-	3	S (21.6)	AHG99684
5	1039.5120	1039.5199	49	^47^ QQFLTHQR^54^	Gln→pyro-Glu	3	S (21.6)	AHG99684
6	1070.4860	1071.5210	29	^43^QQFQTHQR^50^	-	2	S (24.2)	AHG99683
7	1054.4988	1054.4945	57	^43^ QQFQTHQR^50^	Gln→pyro-Glu	2	S (24.2)	AHG99683

^1^Score-values calculated by Mascot [Score = −10 × Log (*p*)] express the probability *p* that a match of calculated and experimental mass is by chance; a score of 30, for example, accounts for *p* ≤ 0.001

^2^The numbers before and after each sequence indicate the residue positions (relative to Met^1^) in the corresponding preprosequences (the accession numbers of these sequences are shown in the last column); underlined residues are modified as shown in the column on the right

^3^Small and large chains are indicated by S and L, respectively, and these chains correspond to the protein bands with apparent molecular masses of 4.1 and 8.1 kDa as shown in Fig. 6; sequence coverage is the percentage of the corresponding chains covered by matching peptides and is indicated in parenthesis

The N-terminal sequence of the 8.1 kDa polypeptide was determined to be RPAIQRCCQQLRNIQPRCR. This sequence corresponds to a 19-residue segment, from Arg^93^ to Arg^111^ (relative to Met^1^) in the primary structure of prepro*Mo*-CBP_3_–3, as highlighted in Fig. 2, thus proving the identity of the 8.1 kDa band. Only one residue was identified after each Edman degradation cycle, suggesting that isoform 3 is present at higher levels compared to the other isoforms. Assuming that the other 3 *Mo*-CBP_3_ isoforms are processed at the same site, the N-terminal residues of their large chains were presumed to be Pro^93^ (isoforms 1 and 4) and Gln^92^ (isoform 2). Assuming that the processing site at the C-terminal end of pro*Mo*-CBP_3_ is the same as that observed for proMabinlin-II, the length of the large chain of the 4 *Mo*-CBP_3_ isoforms should be 68 aa residues (*Mo*-CBP_3_–1 and *Mo*-CBP_3_–4: ^93^PPT···RQQ^160^; *Mo*-CBP_3_–2: ^91^QPA···RQQ^158^; *Mo*-CBP_3_–3: ^93^RPA···GQQ^160^). When the 8.1 kDa band resolved by tricine-SDS-PAGE was submitted to in-gel digestion and the products were analyzed by LC-ESI-MS/MS, 3 peptides were identified (Table 2, peptides 1–3). The sequences of these peptides matched exactly with a specific segment in the supposed primary structures of the large chain of *Mo*-CBP_3_–2 (1 peptide) and *Mo*-CBP_3_–3 (2 peptides). These findings provided further evidence on the identity of the 8.1 kDa band. Therefore, the molecular masses of the large chain were calculated to be 7,756.85 (*Mo*-CBP_3_–1), 7,837.81 (*Mo*-CBP_3_–2), 7,794.93 (*Mo*-CBP_3_–3) and 7,772.88 Da (*Mo*-CBP_3_–4). These values agree with the apparent molecular masses for the small (4.1 kDa) and large (8.1 kDa) chains of *Mo*-CBP_3_ as determined by tricine-SDS-PAGE (Fig. 6).

The total molecular masses of the isoforms were then calculated to be 11,786.8 (*Mo*-CBP_3_–1), 11,881.8 (*Mo*-CBP_3_–2), 12,197.1 (*Mo*-CBP_3_–3) and 11,816.8 Da (*Mo*-CBP_3_–4). The native molecular mass of *Mo*-CBP_3_, as estimated by gel filtration chromatography, was 14.3 kDa [8]. Because the behavior of a protein when it is submitted to gel filtration is also influenced by its shape [63], the value of *ca* 14 kDa was a good approximation when compared with the total molecular masses of the *Mo*-CBP_3_ isoforms (~11.8–12.2 kDa) calculated from their primary structures. Furthermore, the isoelectric points calculated from the sequences of the *Mo*-CBP_3_ isoforms were 11.7 (*Mo*-CBP_3_–1 and *Mo*-CBP_3_–4) and 11.6 (*Mo*-CBP_3_–2 and *Mo*-CBP_3_–3), which are in good agreement with the experimentally determined value of 10.8, which was previously reported [8].

When the reduced and alkylated *Mo*-CBP_3_ was submitted to in-solution tryptic digestion and the products were analyzed by LC-ESI-MS/MS, 14 peptides were identified (Table 3). Thirteen of these peptides matched exactly with specific segments in the primary structures of the precursors of *Mo*-CBP_3_–2 (6 peptides) and *Mo*-CBP_3_–3 (6 peptides). Moreover, the sequence of 1 peptide matched a segment shared by the precursors of *Mo*-CBP_3_–1 and *Mo*-CBP_3_–4. Therefore, the peptides identified from in-gel and in-solution digestions confirmed that the cloned cDNAs code for *Mo*-CBP_3_ and that at least 3 of the 4 identified isoforms are expressed during *M*. *oleifera* seed development.

**Table 3:** Amino acid sequences of *Mo*-CBP_3_ peptides identified by LC-ESI-MS/MS from in-solution tryptic digestions of the purified protein.

	Mass (Da)							
Peptide	Experimental	Calculated	Score^1^	Sequence^2^	Modification	Isoform	Chain and coverage (%)^3^	GenBank accession number
1	2129.1028	2129.1090	85	^115^QAVQLTHQQQGQVGPQQVR^133^	-	2	L (27.9)	AHG99683
2	2112.0772	2112.0825	104	^115^ QAVQLTHQQQGQVGPQQVR^133^	Gln→pyro-Glu	2	L (27.9)	AHG99683
3	842.4010	842.4069	37	^103^NISPPCR^109^	-	2	L (10.3)	AHG99683
4	1071.5222	1071.5210	41	^43^QQFQTHQR^50^	-	2	S (24.2)	AHG99683
5	1054.4614	1054.4945	53	^43^ QQFQTHQR^50^	Gln→pyro-Glu	2	S (24.2)	AHG99683
6	1387.6468	1387.6528	47	^41^CRQQFQTHQR^50^	-	2	S (30.3)	AHG99683
7	1056.5442	1056.5465	57	^47^QQFLTHQR^54^	-	3	S (21.6)	AHG99684
8	1039.5144	1039.5199	53	^47^ QQFLTHQR^54^	Gln→pyro-Glu	3	S (21.6)	AHG99684
9	2389.1356	2389.1341	51	^140^LASNIPAICNLRPMSCPFGQQ^160^	Oxidation	3	L (30.9)	AHG99684
10	2099.1036	2099.0984	114	^117^QAVQLAHQQQGQVGPQQVR^135^	-	3	L (27.9)	AHG99684
11	2082.0448	2082.0719	113	^117^ QAVQLAHQQQGQVGPQQVR^135^	Gln→pyro-Glu	3	L (27.9)	AHG99684
12	1471.7077	1471.7136	57	^99^CCQQLRNIQPR^109^	-	3	L (16.2)	AHG99684
13	1327.6732	1327.6740	74	^144^IPAICNLQPMR^154^	Oxidation	4	L (16.2)	AHG99682
14	2568.2206	2568.2252	50	^117^QAVQSAQQQQGQVGPQQVGHMYR^139^	Oxidation	1/4	L (33.8)	AHG99682

^1^Score-values calculated by Mascot [Score = −10 × Log (*p*)] express the probability *p* that a match of calculated and experimental mass is by chance; a score of 30, for example, accounts for *p* ≤ 0.001

^2^The numbers before and after each sequence indicate the residue positions (relative to Met^1^) in the corresponding preprosequences (the accession numbers of these sequences are shown in the last column); underlined residues are modified as shown in the column on the right

^3^Small and large chains are indicated by S and L, respectively, and these chains correspond to the protein bands with apparent molecular masses of 4.1 and 8.1 kDa as shown in Fig. 6; sequence coverage is the percentage of the corresponding chains covered by matching peptides and is indicated in parenthesis

### Amino acid sequence identity between *Mo*-CBP_3_ and typical 2S albumins

**Fig 7:**
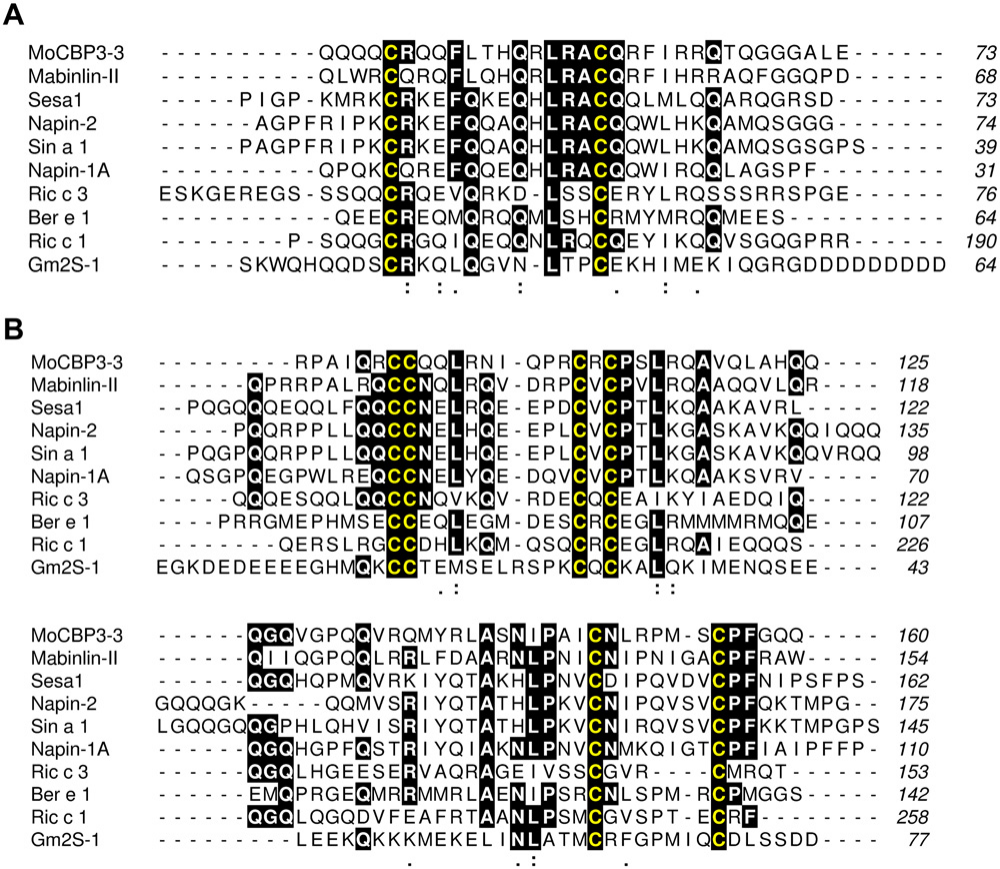
Alignments of the amino acid sequences of the small (A) and large (B) chains of *Mo*-CBP_3_–3 to the corresponding sequences of representative 2S albumins. The sequences of the small and large chains of *Mo*-CBP_3_–3 were deduced from the cognate cDNA (this work). All the other sequences were retrieved from the GenBank database and are as follows (source species and sequence accession numbers are shown in parenthesis): Mabinlin-II (*Capparis masaikai*; P30233), Sesa1 (*Arabidopsis thaliana*; P15457), Napin-2 (*Brassica napus*; P01090), Sin a 1 (*Sinapis alba*; P15322), Napin-1A (*B*. *napus*; P24565), Ric c 3 (*Ricinus communis*; P01089), Ber e 1 (*Bertholletia excelsa*; P04403), Ric c 1 (*R*. *communis*; P01089), and Gm2S-1 (*Glycine max*; P19594). The alignments were performed using the program Clustal Omega. Positions containing the same residue in at least 6 sequences are shaded, and the Cys residues are highlighted in yellow. Sites containing residues with side chains that have strongly (:) or weakly (.) similar properties, scoring > 0.5 and ≤ 0.5 in the Gonnet PAM 250 matrix [93], respectively, are also indicated. The numbers of the residues relative to Met^1^ are shown on the right side of each sequence. The alignment was rendered using the program ALINE [94].

The primary structures of the small and large chains of one *Mo*-CBP_3_ isoform (*Mo*-CBP_3_–3) were aligned to the corresponding aa sequences of representative 2S albumins from species belonging to diverse plant families such as Capparaceae (Mabinlin-II from *C*. *masaikai*), Brassicaceae (Sesa1 from *Arabidopsis thaliana*, Napin-1A and Napin2 from *Brassica napus*, and Sin a 1 from *Sinapis alba*), Euphorbiaceae (Ric c 1 and Ric c 3 from *Ricinus communis*), Lecythidaceae (Ber e 1 from *Bertholletia excelsa*) and Fabaceae (Gm2S-1 from *Glycine max*). The mean percentage sequence identities between the small and large chains of *Mo*-CBP_3_–3 and the corresponding chains of other 2S albumins were approximately 41.5% (33.3% to 62.9%) and 36.7% (21.4% to 53.5%), respectively. Pairwise comparisons among all the sequences revealed mean sequence identities of ~37.6% (small chain) and 36.7% (large chain). However, sequence identities as low as 14.8% (between the small chains of Mabinlin-II and Ric c 3) and 17.8% (between the large chains of Mabinlin-II and Gm2S-1) were observed. Indeed, in the multiple alignments, only 3 and 6 aa residues were conserved in the small and large chains of the compared proteins, respectively (Fig. 7). The conserved residues include the cysteines of the 8CM and one Leu residue in the small chain. These numbers correspond to ~11.1% and 10.7% of the aligned residues of the small and large chains, respectively.

### Relationship between *Mo*-CBP_3_ and the proteins MO2X, MoL and cMoL from *M*. *oleifera*

**Fig 8:**
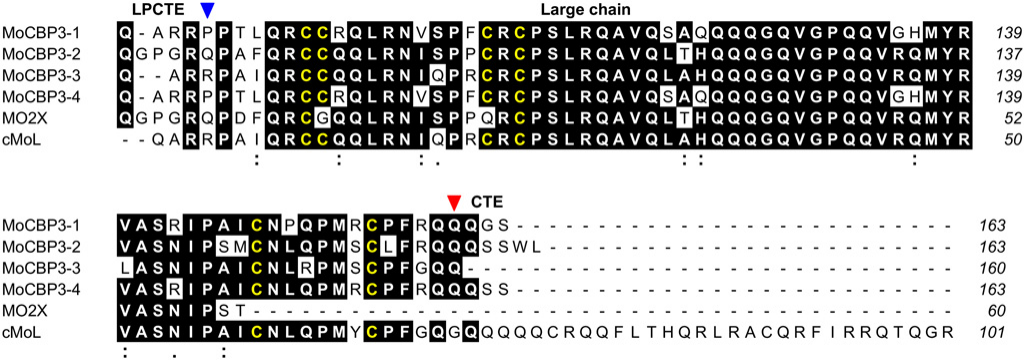
Multiple alignment of the amino acid sequences of a segment of the *Mo*-CBP_3_ precursors with the polypeptide chains of MO2X and cMoL. The amino acid sequences of a segment of the precursors of *Mo*-CBP_3_ were aligned to the primary structures of MO2X (GenBank accession number P24303) and cMoL [65] using Clustal Omega. Positions containing the same residue in at least 4 sequences are shaded and the Cys residues are highlighted in yellow. Sites containing residues with side chains that have strongly (:) or weakly (.) similar properties, scoring > 0.5 and ≤ 0.5 in the Gonnet PAM 250 matrix [93], respectively, are also indicated. The linker peptide C-terminal end (LPCTE), the large chain and the C-terminal extension (CTE) of the *Mo*-CBP_3_ precursors are labeled. The putative processing site of the CTE is indicated by a red triangle, whereas the N-terminal residue of the large chain of *Mo*-CBP_3_, as identified by Edman degradation, is indicated by a blue triangle. The numbers of the *Mo*-CBP_3_ residues relative to Met^1^ are shown on the right side of each sequence. The alignment was edited using the program ALINE [94].

As shown above, *Mo*-CBP_3_ is a typical 2S albumin composed of a small (~4 kDa) chain and a large (~8 kDa) chain linked by disulfide bonds (Fig. 6). Other earlier reported proteins from the same plant source include MO2X, MoL and cMoL. MO2X refers to the flocculent-active proteins MO2.1 and MO2.2. MO2.1 and MO2.2 are natural variants (they differ by a single residue) of a small protein composed of a single polypeptide chain with 60-aa residues and apparent molecular mass of ~6.5 kDa. The 6.5 kDa monomer associates into homodimers of ~14 kDa that are stabilized by disulfide bonds [4], [6]. On the other hand, MoL is a *M*. *oleifera* seed lectin that agglutinates human as well as rabbit erythrocytes and has a binding specificity to glycoproteins. MoL is a homodimer (~14 kDa) in which the monomers (~7 kDa) are linked by disulfide bonds [64]. More recently, a new coagulant lectin (cMoL) that agglutinates human and rabbit red blood cells was purified from *M*. *oleifera* seeds. cMoL is composed of a polypeptide chain of ~11.9 kDa that forms homotrimers of *ca* 30 kDa [65]. The primary structures of MO2X [4] and cMoL [65] have been determined, and the possible relationship between *Mo*-CBP_3_ and these other proteins was then investigated. As depicted in Fig. 8, the polypeptide chain of MO2X aligned with the large chain of *Mo*-CBP_3_ isoforms and the sequence identity between them ranged from 70.6 to 93.1%. Indeed, the MO2X polypeptide is a shorter version of the *Mo*-CBP_3_ large chain where the last 13 C-terminal residues are truncated. The amino acid sequence of cMoL also aligned with the large chain of *Mo*-CBP_3_ isoforms (Fig. 8). The sequence identity between them varied from 75.7 to 94.2%, but in this case, the cMoL polypeptide corresponds to a longer large chain containing extra residues at the C-terminal end. Although the primary structure of MoL is unknown, its subunit composition is clearly different in comparison to that of *Mo*-CBP_3_, and contrary to MoL, *Mo*-CBP_3_ does not have hemagglutinating activity [8]. Therefore, although the primary structures of the MO2X and cMoL chains share sequence identity with the large chain of *Mo*-CBP_3_ isoforms, *Mo*-CBP_3_ is distinct from these other proteins previously reported in *M*. *oleifera* seeds.

## Discussion

Prolamins, globulins (7–8S and 11–12S) and 2S albumins are the main classes of seed storage proteins. Most 2S albumins are heterodimeric proteins composed of one small chain (~4 kDa) and one large chain (~8–9 kDa) linked by disulfide bonds. The small and large chains are synthesized as single precursors that undergo proteolytic cleavages during their maturation. These post-translational cleavages include the removal of an internal peptide located between the segments corresponding to the small and large chains as well as the loss of N- and C-terminal extensions [49], [57]. Plant genomes often contain several 2S albumin genes which are usually intronless and organized in tandem. As a consequence, 2S albumins commonly occur as a mixture of isoforms corresponding to different gene products [66], [67].

In the present study, 4 cDNAs encoding isoforms of the chitin-binding protein *Mo*-CBP_3_ from *M*. *oleifera* seeds were obtained. A comparative analysis of the deduced aa sequences demonstrated that *Mo*-CBP_3_ is indeed a member of the 2S albumin family, as evidenced by the presence of the typical 8CM domain. Similar to most 2S albumins, *Mo*-CBP_3_ is composed of a small chain (~4.1 kDa) and a large chain (~8.1 kDa) linked by disulfide bonds. The small and large chains of *Mo*-CBP_3_ are presumably produced from the proteolytic processing of the preproproteins encoded by the corresponding cDNAs. Moreover, *Mo*-CBP_3_ exists as a mixture of isoforms, corresponding to different mRNA products, as detected by LC-ESI-MS/MS analysis.

2S albumins and other classes of storage proteins are an important source of amino acids during seed germination and early seedling growth [68]. However, diverse biological activities have been described for these seed storage proteins over the last two decades. For example, napins and napin-type 2S albumins from different Brassicaceae species (*A*. *thaliana*, *B*. *napus*, *B*. *rapa*, *S*. *alba* and *Raphanus sativus*) exhibited a broad spectrum of antifungal activity against plant pathogenic fungi [60], [69]. More recently, a typical 2S albumin from the seeds of the passion fruit (*Passiflora edulis*, Passifloraceae) was shown to inhibit the growth of the phytopathogens *Fusarium oxysporum* and *Colletotrichum lindemuthianum in vitro* [70]. Experimental evidence suggest that 2S albumins exert their growth inhibitory activities through the permeabilization of fungal membranes [71], [72]. Some antifungal 2S albumins, such as those from pumpkin (*Cucurbita maxima*, Cucurbitaceae) and *Putranjiva roxburghii* (Putranjivaceae), have also been shown to possess DNase and RNase activities [73–75].

It was shown that *Mo*-CBP_3_ possesses *in vitro* antifungal activity against the phytopathogenic fungi *F*. *solani*, *F*. *oxysporum*, *C*. *musae* and *C*. *gloeosporioides* [8]. These authors also showed that *Mo*-CBP_3_ caused permeabilization of *F*. *solani* cells and appeared to interfere with the plasma membrane H^+^-ATPase of the target cells. Identification of *Mo*-CBP_3_ as a typical 2S albumin extends the spectrum of seed storage albumins with antifungal properties, thus reinforcing the hypothesis that these proteins are involved in plant defense. Furthermore, *Mo*-CBP_3_ is highly thermostable, as it retained its antifungal activity after treatment at 100°C for 1 h [8]. *Mo*-CBP_3_ is also resistant to pH changes, and its antifungal activity is maintained at pH values ranging from 4.0 to 10.0 [9]. Mabinlin-II, the closest known homologue of *Mo*-CBP_3_, comprises five α-helices that are closely packed in a compact structure that is stabilized by four disulfide bonds [76]. This compact fold adopted by the small and large chains held together by disulfide bonds is a characteristic feature of the 2S albumins and makes them extremely stable and resistant to heat as well as proteolytic degradation [57], [77]. The three-dimensional structure of *Mo*-CBP_3_ is likely to be very similar to the structure of Mabinlin-II, and this could explain the resistance of *Mo*-CBP_3_ antifungal activity to temperature and pH variations. Indeed, circular dichroism (CD) spectroscopy analysis has shown that the CD spectral shape of *Mo*-CBP_3_ did not change from pH 2.0 to pH 12.0, and after heat treatment at 100°C for 1 h, its CD spectra showed only minor alterations [9].

*Mo*-CBP_3_ binds to chitin, and this property has been exploited to purify the protein from the albumin fraction of *M*. *oleifera* seeds by chitin affinity chromatography [8]. Chitin is a linear homopolymer of β-(1,4)-linked *N*-acetyl-D-glucosamine residues that constitutes the most important structural component of the cell walls of fungi and the exoskeleton of insects [78]. This polysaccharide is also found in the peritrophic matrix, a chitin and glycoprotein layer that lines the midgut of most invertebrates [79]. To the best of our knowledge, this chitin-binding ability of *Mo*-CBP_3_ is a new property that was not previously reported for a typical 2S albumin, although other classes of seed storage proteins such as vicilins (7–8S storage proteins) are known to bind chitin *in vitro* and chitinous structures *in vivo* [80], [81]. These chitin-binding vicilins also inhibit fungal growth as well as larval development of insects, and these deleterious effects have been attributed to their interactions with the chitinous structures of fungal cell walls and the insect's midgut [82–86].

However, 2S albumins and 7–8S vicilin-type seed storage proteins are not related to each other. Vicilins are homotrimers of ~150–190 kDa that lack Cys residues, and each subunit (~40–80 kDa) contains two copies of the cupin superfamily domain, which adopts a conserved β-barrel structure [49], [87].

In plants, the stereotypical chitin-binding domain is the hevein domain, which was first discovered in the latex of rubber tree (*Hevea brasiliensis*) [88]. The hevein domain has 30 to 43 aa residues organized around a conserved core with 3–5 disulfide bonds, and its structure consists of an antiparallel β-sheet containing two to four strands with helical regions on either side [89–91]. Hevein binds to chitin using a carbohydrate-binding site located on the surface of the molecule [92]. The 2S albumin fold and the hevein domain do not share any structural resemblance; therefore, the mechanism by which *Mo*-CBP_3_ interacts with chitin is yet to be determined.

Excluding the conserved Cys residues of the 8CM, the primary structures of the small and large chains from *Mo*-CBP_3_ and other 2S albumins have low sequence identity, as shown in Fig. 7. Despite this large variation in their amino acid sequences, these 2S albumins adopt a similar five-helix fold, as revealed by an analysis of the three-dimensional structures available to date [57]. In these structures, the network of disulfide bonds maintains the structural scaffold of conserved helical regions, which are connected by variable loops. It has been hypothesized that these variable segments have evolved independently, rendering the 8CM domain as a versatile structure that can accommodate different functionalities [53]. *Mo*-CBP_3_ thus represents one example of a protein containing the 8CM structural scaffold that has evolved a specific function, i.e., the ability to bind to chitin.

The question of whether other 2S albumins have chitin-binding properties deserves further investigation. Nevertheless, the identification of a chitin-binding protein as a typical 2S albumin supports the view that members of this family are multifunctional proteins exhibiting a spectrum of potentially exploitable biological activities.
